# Medical and Philosophical Intelligence

**Published:** 1818-11

**Authors:** 


### MEDICAL AND PHILOSOPHICAL INTELLIGENCE.

THE last Number of the *Medico-Chirurgical Journal* contains a relation of an interesting case of *Disease of the Brain*, by Dr. JOHNSON (the editor). We shall endeavour to convey to our readers the ideas of most importance respecting it, but our account must necessarily be very imperfect: the case is too long to transcribe wholly, and the writings of Dr. Johnson will not admit of abridgment. We are not able, in them, "incomptis allinere atrum transverso calamo signum," and shew "quantula sint corpuscula."

A military officer, about thirty years of age, in the summer of the year 1813, experienced a *coup de soleil*, from using very violent exercise under a meridian sun, in ascending a high mountain at Alicant in Spain. A short time after this, he fell down in a fit at Carthagena, from which he was recovered by the abstraction of four pounds of blood, and very active purgatives. Previously to this period, he had for many years been subject to nasal hæmorrhages, which entirely ceased after the *coup de soleil* at Alicant. He became gloomy, irritable in temper, and complained of pain in the left side of the head and about the occiput. He came to England, and soon after suffered frequent epileptic paroxysms. The pain in the head increased with the violence of the epileptic attacks; his intellects became impaired, and the chylopoietic functions much deranged. He had been repeatedly cupped, blistered, and purged; a seton had been made in the neck; and he had undergone a mercurial course, with little advantage.

In August, 1815, he consulted Dr. Johnson: the principal symptoms then were—prominence and suffusion of the eyes; a countenance indicative of fatuity and great anguish; the speech was stammering, and the memory much impaired. He complained of excruciating, but not constant, pain in the left side and back part of his head. The pupils were large, but contracted freely; the pulse was natural, the tongue clean, the appetite good, and the plumpness of the body little diminished; but the stools were dark-coloured, and very offensive.

Dr. Johnson prescribed a continuance of the antiphlogistic measures, and the tincture of cantharides, with a view of producing a determination of blood to the urinary organs. Under this treatment he seemed to amend, and was able to walk three miles every day; but, as winter approached, he became much worse. At length he tottered in his walk, and became confined to the house. He suffered severely from cramp in different parts of the body, and was subject occasionally to involuntary discharges of urine and fæces. About the beginning of July, 1816, the pains in the head became periodical, coming on about the hours of two or three in the morning, with excruciating agony, after several hours' sound sleep. During the day, he complained of no pain whatever; but was stupid, morose, and taciturn. His appetite continued good; but his bowels were constipated, unless when taking purgative medicines. Arsenic was then given to as great an extent as prudence would permit, and blisters were kept open on the back of the neck, without effect. All this time the vascular system was perfectly tranquil.

Hemiplegia of the right side now took place, continued a few days, and then suddenly disappeared. Hyoscyamus and opium were had recourse to, for the purpose of alleviating the paroxysms of agony, but with little or no benefit.

On the 20th of July, he ate heartily, spoke rationally, and walked up to bed himself at ten o'clock. He slept as soundly and tranquilly as ever he had done in his life, till two, when, as usual, he awoke in agonizing pain. He started up in bed, put his hand to his forehead, cried in a tremulous tone of voice—" Oh, my head!" and instantly expired.

The body was examined after death; when the cranium was observed to be remarkably thin, in some places not thicker than a shilling. The dura mater exhibited nothing unusual on its external appearance: it was so intimately united on the left side with the pia mater and the substance of the brain, that they could not be separated from each other. The right hemisphere of the brain was much softer than natural; the left was of a brown colour, " felt quite rotten," and, as it were, nearly dissolved. The instant the knife was applied to make the *sectio ovalis* on this side, a stream of clear colourless fluid gushed out, to the distance of a foot from the wound, and continued to flow till more than half a pint was collected. The left lateral ventricle extended the whole length of the hemisphere; its parieties were condensed and tough; the foramen of Monro was half an inch in diameter; an ounce or more of water was taken from the right ventricle. Adhesion of the cerebellum to the surrounding parts, and thickening of the tentorium and other membranes, was observed. An abscess was found between the lobes of the cerebellum, the sac of which was an inch and a half long by three quarters of an inch in breadth; some yellow tenacious matter was contained in it. The left lateral sinus was completely converted into, or filled up with, a solid body of a semi-cartilaginous texture, and brown colour. The dura mater was, about this part of the skull, a quarter of an inch in thickness, and nearly of a cartilaginous hardness. The coverings of the medulla oblongata, and medulla spinalis, as far down as a knife would reach, were diseased and thickened.

We can only transcribe the following passage from the judicious reflections of the author on the above interesting case:—

" Had the cephalalgia, which appeared at the beginning and continued to the end, been early met by rigid abstinence from animal food and fermented liquors,—by general and local blood-letting, repeated at longer or shorter intervals,—by free evacuations from the bowels,—by a slight and long-continued mercurial action on the absorbent system,—by tranquillity of mind and body,—what a tragic scene might have been prevented!"

---

The use of *Prussic Acid*, as a medicine, is beginning to receive that degree of consideration from physicians, so powerful a remedy appears to merit; but much variation of opinion exists respecting its qualities, and the extent to which it may be exhibited. This has engaged the attention of M. Robiquet, who has published a Memoir on the subject: he very properly observes, that our endeavours to obtain a medicinal preparation identical in point of strength, should be proportionate to its efficacy; this being the most certain means to prevent accidents from its use, and to save a valuable remedy from falling into discredit.

Prussic acid is certainly one of those which, on that principle, must engage the particular attention of pharmaceutists. M. Majendie has obtained, from the use of this acid, beneficial effects too evident and important to be neglected by physicians, and the more so as they have been in cases of a disease in which all other medicines were absolutely useless. But, as this eminent physiologist observes, there probably does not exist a more deleterious substance than this, when it is in a state of purity; it must, therefore, be employed sufficiently diluted with water. M. Majendie used that obtained by the process of Scheele; but it cannot, by this method, be obtained always the same in point of strength. The lixivium of cyanuret *(prussiate)* of mercury may be more or less concentrated; the hydrogen, produced on the solution of the iron, combines with the most pure portion of the acid, and in quantities which vary according to existing circumstances: it cannot, therefore, be obtained, by this means, of the same precise and determinate strength in different processes. M. Robiquet first employed the crystallized cyanuret of mercury with the addition of a determinate proportion of water, sulphuric acid, and iron filings; and then proceeded to its decomposition by the heat of a water-bath. By these means, he obtained an acid more concentrated than, but equally variable with, that produced by the process of Scheele. He next had recourse to the mode pointed out by Proust, which consists in decomposing cyanuret of mercury by sulphuretted hydrogen: this plan appeared to possess many advantages, because, by adding always the same quantity of water to the cyanuret, it might be expected that an acid of equal density might be obtained; but he was obliged to relinquish this process, for many reasons. In the first place, he could not completely free the acid from the excess of sulphuretted hydrogen, neither with litharge nor prussiate of copper; and what more surprised him was, that the acid was of greater density than water: it would appear that it retained a small proportion of mercury.

He lastly had recourse to the method prescribed by M. Gay-Lussac. He adapted a tube, containing some small fragments of marble and fused muriate of lime, to a small tubulated retort; the glass tube terminated in another of lesser diameter, which was inserted into a bottle, to which it was fitted by grinding; this was surrounded by a cooling mixture. He introduced some prussiate of mercury into the retort, to which a quantity of hydrochloric *(oxy-muriatic)* acid, somewhat more than sufficient to cover it, was added. The mixture was then gently heated, so that the hydro-cyanic acid might remain in contact for a considerable length of time with the carbonate and muriate of lime, to free it, in a certain degree, from the hydrochloric acid and water. The product obtained was 0,7 in density; that of Scheele is rarely less than 0,9. The hydro-prussic acid, obtained by the above process, was reduced to the same density as that of Scheele, by diluting it with two parts of water. For conducting this process with more facility, the acid may be received into a graduated phial, so that the necessary quantity of water may be added, without pouring it into another vessel.

Our readers who are versed in chemistry will perceive that the above preparation is not *pure* prussic acid, but a combination of that substance with water; it is also evident that it will, even when procured by this method, be variable in point of strength, according as the vapour may be retained for a greater or lesser length of time over the muriate of lime. These objections may, however, be obviated, by determining on a certain degree of specific gravity, to which the acid may be diluted, and according to which it should be generally directed for use.

To obtain prussic acid in a state of absolute purity requires so much care and trouble, and is so highly dangerous a process to the operator, that few chemists, we believe, would venture to attempt it: it has, however, been effected by Mr. H. C. Jennings. It is difficult to form an adequate idea of the power of this deleterious substance, without having witnessed its effects. A particle applied to the nose of a large Newfoundland dog, (the cuticle being entire,) produced instant death. Nothing, indeed, can be imagined more simultaneous than the contact of the poison and the absolute death of the animal.

---

That species of *Spinal Distortion*, which appears solely to depend on a divergence of the axes of the bones of the vertebræ from the centre of gravity as a consequence of rickets in infancy, but which, when once it has taken place, generally continues to increase, according to the laws of mechanics, until the period of puberty, is now generally treated in the public hospitals in London, with success, by mere confinement of the patient to the horizontal position on an unyielding surface. Medicines calculated to remedy any derangement of the general system, with which it may be accompanied, are occasionally had recourse to at the same time. But the use of caustic issues, setons, perpetual blisters, &c. formerly employed with so little discrimination in spinal distortion, is now generally abolished in the treatment of that species to which we allude. The period required to remedy the derangement is certainly long,—frequently two or three years; but, if the regimen of the patient be judiciously ordered, his health does not suffer material injury.

---

A DISPOSITION appears to be manifesting itself in some surgeons, particularly those engaged in public practice, to introduce the *actual cautery* among our modern armamenta; and, although the aphorism of Hippocrates, ὅσα σίδηρος οὐκ ἰηται, πῦρ ἰηται, will frequently be found true, yet we fear this remedy can never be commonly employed, in consequence of the strong prejudices that exist respecting it among patients in general, arising from the groundless dread they have of its extreme severity. Amongst a few instances, in which it has been used within our observation, we may mention one, where it was applied with apparent advantage to the surface, remaining after the removal of an osseous tumor springing from the alveolar processes of the lower jaw, to destroy the disposition of the parts to the reproduction of the disease.

---

THE *Pyrola umbellata* (an account of which was inserted in the 214th Number of our Journal) is beginning to engage the attention of medical practitioners. It is, indeed, the most powerful direct vegetable diuretic with which we are acquainted. In that species of dropsy which has succeeded to acute diseases, and which seems to arise from increased action of the exhalant arteries, or inflammation of the serous membranes, it has been found eminently beneficial, after the acute stage of the disease has passed away. In combination with other remedies, it has been advantageously employed in various other species of that disease.

---

A NEW instrument has been introduced into medical science at Paris, which, from the favourable report it obtained on being submitted to the Academy of Sciences, would appear to be somewhat more than a chimerical improvement.

Dr. Laennec, physician to the Necker Hospital, supposed it likely that the various sounds which are formed in the interior of the body, as in the breast, &c. might become, from the variation induced on them by disease, indications of the state of health; and that the sounds produced by the action or motion of any particular organ, as of the heart or lungs, would point out any change in the state of that organ; and, taking advantage of the superior conducting power of solid bodies with regard to sound, he formed an instrument which should convey these indicatory sounds more readily and distinctly to the ear. This instrument is a cylinder of wood, which in some cases, according to the nature of the examination, is solid; in others, perforated lengthways by a canal; and in others, hollowed like a horn.—*Annales de Chimie.*

---

*Change of Temperature of the Boa Constrictor.*—IT is, perhaps, not generally known that the boa constrictor of Linnæus changes its temperature as that of the atmosphere becomes altered; but that such is the case appears from the observations of Mr. Wilford. During his residence in Africa, he purchased one of these animals of a Mandingo man: these people occasionally bringing them to Sierra Leone for sale, under the name of tame snakes.* It was three feet four inches long, and grew an inch in a year and a quarter. It lived on rice-flour, and this was the only article of food he ever observed it to eat; it drank about a table-spoonful of water every other day, and evacuated the intestines seldom more than once a week; the urine was voided once in eight or ten days, in quantity about an ounce. Occasionally washing in water seemed to prove highly refreshing and beneficial. Approaching the north, on Mr. Wilford's return to England, the snake became more and more torpid, and at length died; the power of generating heat being too feeble to support the diminution of atmospheric temperature. When the temperature of the atmosphere was $79\frac{1}{4}$ degrees, that of the snake was the same; atmosphere 75, snake 75; atmosphere 72, snake 75; pulsation of the heart, 23 in a minute: this at last became reduced to 20, and at one period 15, in a minute; the animal shortly after died.

It cast its skin in the month of April, and, for three weeks previous to the time, neither ate nor drank. As soon as the old skin was completely thrown off, it resumed its wonted activity.—*Journal of the Royal Institution.*

---

*On the Proteus Anguinus.*—This singular animal, supposed to be a native only of the lake of Sittich in Carniola, whence, in times of flood, it escaped into the *Cirknitzer sea*, and which was generally considered as the larva of some unknown amphibious animal, has lately been the subject of an interesting letter from M. Rudolphi to Professor Linck.

According to Rudolphi, it has recently been discovered in the grotto of St. Madelaine, near Adelsberg, and in some other small lakes or pools in the vicinity, in sufficient abundance to have enabled him to procure fourteen individuals.

The manners and habitudes of this animal bear a great resemblance to those of the salamander. Although the eyes of the proteus are very small, and covered by a skin of considerable thickness, the animal appears very sensible to light; and its motions, when thus exposed, are very brisk; the veins, also, beneath the transparent skin of the animal, became at the same time very turgid. Although capable of enduring so long-continued abstinence that it was supposed to take no solid food, M. Rudolphi found in the stomachs of a few of them the remains of snails and of other small animals.

---

* These animals are not poisonous, and their dangerous power consists merely in their muscular force. In their habits, they are mild and docile, and possess very little of that ferocity and subtleness which characterize the species. Mr. Wilford's snake was his companion, sometimes even at table, for a long period of time.

The irritability and muscular power of the proteus are very feeble. The globular particles of the blood are larger than in any other known animal; and its lungs are a sac much resembling the air-bladder of fishes, which structure, admitting only of a very slow decarbonization of the blood, appears to account for the very singular fact of the conjunct action of lungs and gills in the same animal.

Cuvier, who some years since dissected one of them, demonstrated in it the presence of ovaries; thus rendering the opinion of its being a perfect animal, and not a larva, extremely probable. This observation has been fully confirmed by Rudolphi, who in some individuals detected ovaries, and in others testicles.

---

*New Inflammable Gas.*—Dr. Thomson has discovered a new compound inflammable gas, and has called it, from the nature of its constitution, hydroguretted carbonic oxide. Its specific gravity is ·913, that of common air being 1. It is not absorbed nor altered by water. It burns with a deep-blue flame, and detonates when mixed with oxygen and fired. It is a compound of oxygen, hydrogen, and carbon; and Dr. Thomson considers it as being three volumes of carbonic oxide, and one volume of hydrogen, condensed by combination into three volumes.

---

*Production of Alcohol from Potato-Apples.*—From experiments lately made, it appears that the fruit or apple of the potato yield, by proper treatment, as much alcohol as an equal quantity of grapes. The apples are to be bruised, and fermented with about an eighteenth or twentieth part their weight of some ferment, and then to be distilled.—*Journal of the Royal Institution.*

---

*Alisma Plantago.*—The authorities from Russia are very strong in favour of the use of the alisma plantago as a cure for hydrophobia; and the following description has lately been published, that the use of the plant may become more general.

The alisma plantago grows in water, either in marshes, lakes, or ponds. It has a capillary root, resembling that of an onion. The plant continues under water until the month of June, at the commencement of which, or even during the month of May, in a warm climate, from five to seven long detached sprouts, of a long convex form, shoot from beneath the water. These sprouts have a reddish bark, and are each provided with a pointed, smooth, and deep-coloured leaf. In the month of June, a stalk appears, with a round green root, resembling that of asparagus. This stalk shoots from beneath the water, sometimes with and sometimes without leaves; at the extremity of each of which is a small trefoil flower, of a pale red colour, which afterwards contains the seeds. This plant is in blossom during the whole of the summer season: the latter end of August is the fittest time to gather it. The roots, when gathered, are to be washed, and dried in the shade; and, when used, to be eaten in powder, strewed on bread and butter.

### METEOROLOGICAL JOURNAL,

By Messrs. WILLIAM HARRIS and Co. 50, Holborn, London.

*From the 20th September to the 19th October, inclusive.*

| Day of Month. | Moon. | Rain gauge | THERM. | | | BAROM. | | De Luc's HYGROM. Dry | Damp | | Wind. | | Atmospheric Variation. | | |
|---|---|---|---|---|---|---|---|---|---|---|---|---|---|---|---|
| Sept. | | | | | | | | | | | | | | | |
| 20 | | ,04 | 55 | 62 | 48 | 29·97 | 29·98 | | 10 | 15 | SE | E | Fine | | |
| 21 | | ,09 | 51 | 61 | 50 | 29·61 | 29·40 | | 12 | 19 | E | SE | Fine | Rain | Clo. |
| 22 | ☽ | ,20 | 53 | 64 | 51 | 29·73 | 29·59 | | 11 | 17 | S | S | Fine | Rain | |
| 23 | | ,05 | 56 | 62 | 55 | 29·68 | 29·72 | | 15 | 15 | SSE | SE | Rain | Clo. | Fine |
| 24 | | ,09 | 59 | 62 | 58 | 29·70 | 29·71 | | 13 | 17 | SE | SE | Clo. | Fine | Rain |
| 25 | | ,21 | 59 | 65 | 60 | 29·67 | 29·56 | | 15 | 20 | SE | N | Rain | | Clo. |
| 26 | | ,33 | 63 | 67 | 59 | 29·59 | 29·70 | | 23 | 20 | NE | N | Fine | Clo. | Rain |
| 27 | | ,24 | 60 | 65 | 58 | 29·67 | 29·61 | | 17 | 20 | SE | SSE | Fine | Rain | Fine |
| 28 | | ,17 | 61 | 67 | 54 | 29·68 | 29·70 | | 18 | 13 | SE | SE | Fine | Clo. | Fine |
| 29 | | ,11 | 59 | 66 | 53 | 29·70 | 29·73 | | 12 | 10 | SW | SW | Clo. | Fine | |
| 30 | ☺ | ,07 | 55 | 65 | 57 | 29·70 | 29·57 | | 10 | 11 | SE | SE | Clo. | | |
| Oct. | | | | | | | | | | | | | | | |
| 1 | | | 63 | 67 | 58 | 29·55 | 29·50 | | 7 | 9 | ESE | SSE | Fine | | |
| 2 | | | 62 | 65 | 54 | 29·51 | 29·53 | | 11 | 12 | SE | SE | Fine | | |
| 3 | | ,15 | 60 | 63 | 55 | 29·58 | 29·69 | | 16 | 24 | SE | SE | Fine | | |
| 4 | | ,03 | 59 | 62 | 53 | 29·77 | 29·72 | | 19 | 20 | SE | SE | Fog | Clo. | |
| 5 | | ,04 | 59 | 61 | 53 | 29·59 | 29·54 | | 20 | 17 | SW | SW | Fog | Rain | |
| 6 | | ,17 | 60 | 63 | 50 | 29·54 | 29·54 | | 13 | 14 | SW | WSW | Fine | Rain | Fine |
| 7 | ○ | ,10 | 53 | 59 | 49 | 29·50 | 29·34 | | 14 | 15 | WSW | SW | Fine | Rain | Fine |
| 8 | | ,13 | 53 | 60 | 43 | 29.76 | 29·64 | | 13 | 17 | WSW | W | Fine | Rain | Fog |
| 9 | | ,19 | 43 | 47 | 47 | 29·79 | 29·89 | | 20 | 21 | W | NNW | Fog | Fine | |
| 10 | | | 49 | 53 | 50 | 29·81 | 29·79 | | 17 | 19 | W | W | Fog | Fine | Clo. |
| 11 | | | 53 | 59 | 53 | 29·75 | 29·59 | | 20 | 20 | W | SW | Fog | Rain | Clo. |
| 12 | | ,12 | 59 | 62 | 49 | 29·55 | 29·53 | | 21 | 20 | S | SSW | Clo. | Rain | |
| 13 | | ,22 | 53 | 59 | 49 | 29·72 | 29·91 | | 19 | 15 | S | S | Fine | | |
| 14 | ○ | | 51 | 60 | 53 | 29·86 | 29·96 | | 15 | 16 | S | S | Fine | | |
| 15 | | | 57 | 63 | 53 | 30·00 | 29·96 | | 13 | 19 | S | SSE | Fine | | |
| 16 | | | 59 | 64 | 57 | 29·92 | 29·95 | | 17 | 16 | SSE | SSE | Fine | | |
| 17 | | | 60 | 67 | 55 | 29·97 | 30·00 | | 16 | 17 | SSE | ESE | Clo. | Fine | |
| 18 | | | 57 | 66 | 57 | 30·07 | 30·00 | | 16 | 20 | E | E | Fog | Fine | |
| 19 | | ,03 | 59 | 66 | 55 | 30·00 | 30·00 | | 21 | 19 | S | S | Clo. | | Fine |

The quantity of rain fallen in the month of September is 2 inches and 73-100ths.

### REPORT OF DISEASES.

ON taking a general view of the vast assemblage of maladies presented to our observation in a large metropolis, our attention must necessarily be engaged in a peculiar manner by those combinations of symptoms usually designated by the term Fever,—interesting in themselves, and of the highest importance from their evincing the existence of disease, frequently cognizable with so much difficulty, that we are led to embrace the shadow for the reality. They claim our first and most particular consideration.

Fever, as the chief characteristic of disease, has been less noticed in the last than during the preceding month. Inflammation of the respiratory organs, or of the membranous parts of the body, more or less generally, has usually been evident; and has so clearly pointed out the proper mode of treatment, that few cases of fatal termination have been witnessed. Pneumonia has been frequently followed by chronic inflammation of the mucous membrane of the bronchiæ, sometimes terminating in the formation of purulent matter, but more commonly producing a secretion of viscid phlegm, which, obstructing the air-cells, and preventing the due influence of the air on the blood in its passage through the lungs, has been productive of great debility: this state is usually accompanied with a peculiar sallow bluish tinge of the surface, and great depression of mind. In these cases, the spirit of turpentine, given in doses of from thirty to fifty drops twice a-day, has been found to produce very favourable effects. We have thought that much advantage has resulted from daily ablution of the legs and feet with cold water, after convalescence: it seems to render the patient less susceptible of the influence of the frequent changes of temperature of the atmosphere at this season. Erysipelas has also frequently concurred with fever. It is important that a distinction should be made between this affection as it occurs generally, and to persons previously in health, and that affecting patients confined in an hospital, and which accompanies extensive wounds attended with great irritation. In the former, purgatives and antimonials must not be spared; in the latter, opium, brandy, and other stimulants, are the only means which will induce a favourable termination.

Puerperal fever has lately become rather prevalent; but this no longer causes the alarm in the minds of patients and their attendants that it once produced. The important addition that has lately been made to our knowledge of this disease and the appropriate mode of treatment, has rendered fatal cases of but rare occurrence.

Acute rheumatism has been frequent and severe: in those cases in which it has been principally confined to the extremities, the use of compression, according to the method of Dr. Balfour, has been eminently beneficial. We have not adopted it, in general, until suitable evacuations had been premised.

Ophthalmia has been common during the last week or two. No cases more clearly evince the good effects of stimulants, after the use of proper evacuations, and when the general system is free from disturbance, than those of inflammation of the outer membranes of the eye. Many cases have been cured in a few days by vinum opii, æther diluted with eight or ten parts of water, or other applications of the like nature; which, judging from the result of similar cases when treated by a continuance of bleeding and cold lotions, would have existed as many weeks.

The maladies that usually accompany the approach of winter have hardly made their appearance. Many chronic diseases have, however, assumed a more active form, perhaps from the existence of a greater degree of plethora of the system. That slow, insidious inflammation of the heart, giving rise to enlargement of that organ, which is commonly overlooked in its early stages, has been observed, in several instances, to begin to molest the patient so much, as to make him desirous of obtaining medical aid for feelings, which his acquaintance would endeavour to persuade him solely depend on his imagination. Many patients of this kind evince an irritability and irascibility of disposition, which appear to have been acquired very early in life, and many will observe that they have been subject to throbbing and palpitation of the heart from any unusual mental emotion, for several years. To relieve this irritability, and to remove all the exciting causes of inflammation, are our indications: in many, indeed in the greater number of instances, our means are only temporarily beneficial; but they will also, in some cases, defer the fatal termination for several years. Digitalis and antimony are our agents. We purpose trying the distilled water of laurel in some of these maladies. The advantages derived from the use of prussic acid by M. Majendie (which laurel is known to contain), the favourable accounts published of the effects of laurel-water by M. Cevasco, and the results of some experiments made on ourselves with this substance, lead us to expect much benefit from its use, in all cases in which it is desirable to depress the vital powers.

